# Low-Milliampere CT Fluoroscopy-Guided Percutaneous Drainage Placement after Pancreatic Surgery: Technical and Clinical Outcome in 133 Consecutive Patients during a 14-Year Period

**DOI:** 10.3390/diagnostics12092243

**Published:** 2022-09-16

**Authors:** Christoph G. Trumm, Danilo Hackner, Katharina Badmann, Alexander Crispin, Robert Forbrig, Yigit Ozpeynirci, David Kuppinger, Vera Pedersen, Thomas Liebig, Robert Stahl

**Affiliations:** 1 Institute for Diagnostic and Interventional Neuroradiology, University Hospital, Ludwig-Maximilians-University (LMU), Marchioninistr. 15, 81377 Munich, Germany; 2 Department of General and Visceral Surgery, University Hospital Erlangen, Friedrich-Alexander-University (FAU) Erlangen-Nuremberg, Krankenhausstr. 12, 91054 Erlangen, Germany; 3 Department of Radiology, University Hospital, Ludwig-Maximilians-University (LMU), Marchioninistr. 15, 81377 Munich, Germany; 4 IBE—Institute for Medical Information Processing, Biometry and Epidemiology, Ludwig-Maximilians-University (LMU), Marchioninistr. 15, 81377 Munich, Germany; 5 Department of General, Visceral, Transplantation, and Vascular Surgery, University Hospital, Ludwig-Maximilians-University (LMU), Marchioninistr. 15, 81377 Munich, Germany; 6 Department of Orthopaedics and Trauma Surgery, Musculoskeletal University Center Munich (MUM), University Hospital, Ludwig-Maximilians-University (LMU), Marchioninistr. 15, 81377 Munich, Germany

**Keywords:** technical outcome, clinical outcome, CT-guided drainage, pancreatic resection, fluid collection

## Abstract

(1) Purpose: To retrospectively assess the technical and clinical outcome of patients with symptomatic postoperative fluid collections after pancreatic surgery, treated with CT-guided drainage (CTD). (2) Methods: 133 eligible patients between 2004 and 2017 were included. We defined technical success as the sufficient drainage of the fluid collection(s) and the absence of peri-interventional complications (minor or major according to SIR criteria). Per definition, clinical success was characterized by normalization of specific blood parameters within 30 days after the intervention or a decrease by at least 50% without requiring additional surgical revision. C-reactive protein (CRP), Leukocytes, Interleukin-6, and Dose length product (DLP) for parts of the intervention were determined. (3) Results: 97.0% of 167 interventions were technically successful. Clinical success was achieved in 87.5% of CRP, in 78.4% of Leukocytes, and in 87.5% of Interleukin-6 assessments. The median of successful decrease was 6 days for CRP, 5 days for Leukocytes, and 2 days for Interleukin-6. No surgical revision was necessary in 93.2%. DLP was significantly lower in the second half of the observation period (total DLP: median 621.5 mGy*cm between 2011–2017 vs. median 944.5 mGy*cm between 2004–2010). (4) Conclusions: Technical success rate of CTD was very high and the clinical success rate was fair to good. Given an elderly and multimorbid patient cohort, CTD can have a temporizing effect in the postoperative period after pancreatic surgery. Reducing the radiation dose over time might reflect developments in CT technology and increased experience of interventional radiologists.

## 1. Introduction

The worldwide incidence of pancreatic cancer is rising, particularly among women and populations of 50 years or older [1]. Surgical resection (R0) is the sole curative treatment for patients with the non-metastatic disease [2]. Various types of surgical approaches to pancreatic tumors depending on their localizations are available, e.g., classic pancreaticoduodenectomy (PD, Whipple-Kausch procedure), pylorus preserving pancreaticoduodenectomy (PPPD) and left-sided pancreatic resection (distal pancreatectomy, DP) [3]. In addition, minimally invasive surgical techniques in pancreatic surgery are available [4]. However, pancreatic surgery is demanding since anastomosis of the pancreatic duct is technically challenging. Additional organ resection is often necessary, depending on the type of surgical approach as well as local and systemic tumor spread. Therefore, these operations often result in altered abdominal anatomy and make the patients susceptible to surgical complications such as leaks and fistulas [5].

Consequently, intraabdominal fluid collections frequently occur after pancreatic surgery [6,7]. The most frequent entities causing fluid collections are pancreatic fistula, leak, abscess, and haemato-seroma [7,8]. Postoperative pancreatic fistula (POPF) and abscess formations can involve substantial morbidity and mortality [9]. Computed tomography (CT) is commonly used to characterize fluid collections and to clarify whether additional treatment is required [10].

The most frequently used therapeutic measures are antibiotic treatment in conjunction with percutaneous drainage. The latter allows precise targeting of the fluid collection site and the asservation of an adequate fluid specimen for microbiological characterization and targeted antibiotic therapy [11,12,13]. In the non-surgical armamentarium percutaneous drainage has also proven to be an option for the effective management of pancreatic fistula [14].

Generally, percutaneous drainage under sequential or intermittent CT fluoroscopic guidance is a commonly carried out minimally invasive procedure that is well tolerated and therefore preferred by the patients in comparison to open surgical drainage [15,16]. Technical success rates of percutaneous drainage are very high [11,17,18]. On the other hand, major complications such as hemorrhage or sepsis may follow percutaneous drainage rarely [12,19,20,21,22].

Several studies focused on the clinical outcomes of percutaneous CT-guided drainage (CTD) of intraabdominal fluid collections of varying entities after pancreatic surgery. Most authors underlined high clinical success rates [8,23,24,25,26]. As a limitation, these studies were characterized by comparatively small patient collectives.

Our study aimed to assess technical and clinical results of a large single-center patient cohort with symptomatic fluid collections after pancreatic resection undergoing low-milliampere CT fluoroscopy-guided drainage.

## 2. Materials and Methods

### 2.1. Study Subjects

**Figure 1:**
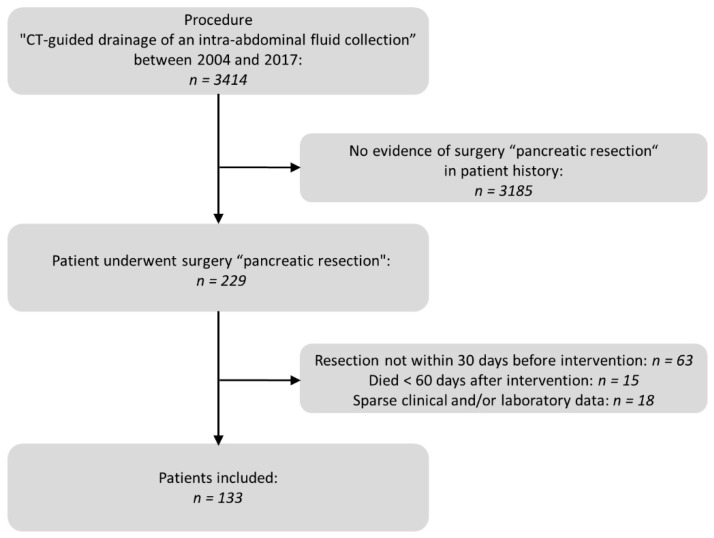
Patient selection process flow chart.

We performed a database search of the Radiology Information System (RIS) for the specific procedure key “CT guided drainage of an intraabdominal fluid collection”. A subsequent full-text search of the corresponding written reports as well as the operations and procedure codes (OPS) of the Hospital Information System (HIS) indicated previous pancreatic resections. We evaluated patients with postoperative fluid collections who had undergone pancreatic resection between 2004 and 2017 and received percutaneous low-milliampere CT fluoroscopy-guided drainage during a maximum postoperative period of 30 days. For reasons of homogeneity of the patient collective, a minimum survival period of 60 days was set. Fifteen patients died before the end of the observation period. In all these cases a correlation between the intervention and death cause could be excluded. Details of the selection process are displayed in Figure 1.

All interventional procedures performed in this study involving humans were in accordance with the Helsinki Declaration of 1964, the ethical standards of the institutional and/or national research committee, and its later amendments or comparable ethical standards. Informed consent by the patients or their legal guardians to undergo CT-guided drainage was usually obtained 24 h before the intervention and in case of emergency immediately prior to the procedure. This retrospective study was approved by the local ethics committee (number 21-0755).

### 2.2. CT Imaging Protocol

All interventions were performed on a 16-slice (Somatom Sensation 16; Siemens Healthineers, Erlangen, Germany), a 64-slice (Siemens SOMATOM 64), or a 128-slice (Siemens SOMATOM Definition AS+/Siemens SOMATOM Definition Edge) CT scanner. The scanners were equipped with the C.A.R.E. Vision application to provide dose-saving techniques during CT fluoroscopic scans. Patients underwent a contrast-enhanced pre-interventional CT scan to examine the exact position and size of the fluid collection. Based on these images the most appropriate access trajectory was identified. If a contrast-enhanced CT scan performed less than 24 h prior to the intervention was available, only a non-enhanced CT scan was conducted.

The choice of drainage catheters with different diameters (Flexima^®^, Boston Scientific Corporation, Marlborough, MA, USA and ReSolve^®^, Merit Medical, South Jordan, UT, USA; respectively) depended on the access path, the assumed consistency of the fluid collection, and the experience of the interventionalist. An unenhanced follow-up CT scan was performed immediately after drainage catheter placement to evaluate the outcome of the intervention with respect to the position of the drainage and potential peri-interventional complications. Images were reconstructed using a soft tissue convolution kernel at a slice thickness of 3 mm.

All procedures were performed by a total of ten different interventionalists who had at least 5 years of experience in interventional radiology.

### 2.3. Analysis of Pre- and Peri-Interventional Period

One board-certified radiologist with more than 10 years of experience in abdominal imaging assessed indications for pancreatic resection, surgical techniques, predominant locations of fluid collection, interventional techniques (Trocar vs. Seldinger technique), number of drainages, diameter of drainage catheters, access trajectory for drainage (transabdominal, transhepatic, transretroperitoneal) and peri-interventional complications (minor, major) according to the SIR criteria [27]. Additionally, complications were evaluated by the Clavien–Dindo classification [28]. Measurements of the mean diameter of fluid collections were taken and fluid collection entities were differentiated.

After the intervention, success in technical outcome was defined as sufficient drainage of the fluid collection (i.e., leaving less than 20% of the fluid collection after aspiration as estimated using the postinterventional CT scan) and the absence of peri-interventional complications [12].

Assessments of inflammatory blood parameters (C-reactive protein (CRP), Leukocytes, and Interleukin-6) were conducted before and after the intervention to detect possible superinfections.

The CT scanner provided the patient with a radiation dose for every interventional procedure using the dose-length product (DLP (mGy*cm)). The analysis comprised DLP of the pre-interventional planning CT scan, the sum of all intra-interventional CT fluoroscopic acquisitions, and of the post-interventional control CT scan. DLP results between 2004–2010 and 2011–2017 were compared.

### 2.4. Analysis of Post-Interventional Period

Patients receiving reoperation within 60 days after surgery due to insufficient drainage of the fluid collections were subject to the study analysis.

According to Bassi et al. [29], the presence of a postoperative pancreatic fistula (POPF) was assumed if the amylase content in the drainage fluid was three times higher than the normal serum level on or after the third postoperative day.

Inflammatory parameters within 30 days after the intervention were extracted from the clinical patient chart. Based on these results, success in postinterventional clinical outcome was defined as either a decrease (>50%) of the initially elevated parameters CRP, Leukocyte count, and Interleukin-6 or by normalization of these elevated parameters within 30 days after the intervention. Additionally, clinical success was defined by the absence of the need for any further surgical procedure related to the intervention. The clinical outcome was subsequently compared with the applied surgical techniques to detect possible causal relations.

The microbiological results of the secretion delivered by the drainage catheters were evaluated. Additionally, post-interventional serum amylase levels were monitored.

Removal dates of each patient’s drainage were registered. The removal of the drainage was based on the patient’s clinical and laboratory response. Follow-up imaging was performed only in patients who were not improving clinically.

### 2.5. Statistical Analysis

Initially, the Shapiro-Wilk test and visual inspection of histograms were used to assess the normality of discrete and continuous data. Normally distributed variables are presented as mean ± standard deviation (sd). Variables with non-normal distribution are displayed as median (25%-; 75%-quartiles).

For binary (e.g., presence of POPF, need for surgical revision) or categorical (e.g., applied surgery technique, visual appearance of the fluid collection) variables, contingency tables were created. The independence of these variables was assessed with Chi^2^- or Fisher exact tests, depending on the size of the respective contingency tables (Fisher exact test for fourfold tables, otherwise Chi^2^-tests). In case of statistically significant results in the Chi^2^ tests or in the post hoc Fisher exact tests Bonferroni corrections were applied.

For analysis of the 30-day post-interventional time course, the values of the blood parameters were log-transformed to achieve normal distribution. Subsequently, generalized linear mixed models (GLMM) were carried out. Fixed effects were given by the number of days after the intervention and the adjustment for the presence of POPF and proof of germs in the drainage fluid. Random intercepts were included by subject ID and repeated by days.

Differences between the radiation exposure in the two time periods were assessed with Mann-Whitney tests for independent samples. Analysis was performed using R (R Core Team, Vienna, Austria (2020). R: A language and environment for statistical computing. R Foundation for Statistical Computing, Vienna, Austria. URL https://www.R-project.org/ version 4.0.2, accessed on 22 Jun 2020). A level of significance of alpha = 0.05 was assumed for all analyses in the study.

## 3. Results

We included 133 patients (61 females; age (median [25%, 75% quartile]) 64 [55, 72] years) having undergone CT-guided drainage following pancreatic resection between 2004 and 2017. Detailed information on the preceding surgical procedures is provided in Table 1. A pancreatico-jejunostomy was performed in 95 (71.4%) cases. In the context of the pancreatic resection, a biliodigestive anastomosis was applied in 94 (70.7%) and a (gastro-)intestinal anastomosis was conducted in 97 (72.9%) of the patients. Supplementary Table S1 shows an overview of the underlying disease in the patient group.

### 3.1. Pre- and Peri-Interventional Analysis

A total of 167 interventions after surgery (mean ± SD: 1.4 ± 0.5 per patient) were performed within a 30-days interval.

Seldinger technique was chosen in 16 (9.6%) procedures whereas the Trocar technique was preferred in 151 (90.4%) cases by the discretion of the interventionalist. The mean number of drainages was 1.3 (SD: ±0.5) per intervention. In total, 208 drainages were inserted. Distribution of drainage diameters was as follows: 6 French (F) in 1 case (0.7%), 7.5F in 3 cases (2.1%), 8F in 66 cases (46.8%), 10F in 53 cases (37.6%), 12F in 14 cases (9.9%), 14F in 3 cases (2.1%) and 20F in 1 case (0.7%). With respect to 67 drainage cases, no information on the diameters was available. The most common access path was through the abdominal wall in 124 procedures (74.3%), followed by a transretroperitoneal approach in 26 drainage placements (15.6%). Detailed information on drainages and intervention techniques is provided in Table 2.

During the observation period, up to three interventions were performed on individual patients. In 103 patients only one intervention was required (77.4%). In four cases three procedures were conducted (3.0%). The amount of interventions did not differ significantly (*p* > 0.05) between the applied surgical techniques (Table 3).

Analyzing the drainage fluid, 59 patients had a POPF according to the biochemical definition. Fistulas were most frequently present after distal pancreatectomy (23 out of 40, 57.5%) and least frequently observed after classic pancreaticoduodenectomy (11 out of 34, 32.4%), which was not statistically significant (*p* > 0.05).

Positive germ detection at first intervention was present in 77 of 133 patients (57.9%). This proportion was significantly (*p* < 0.05) lower in patients with DP (14 out of 40, 35.0%) in comparison to patients where other surgical techniques were applied. However, the presence of POPF, as well as the infection status of the drainage fluid did not significantly (*p* > 0.05) affect the number of interventions performed per patient.

**Figure 2:**
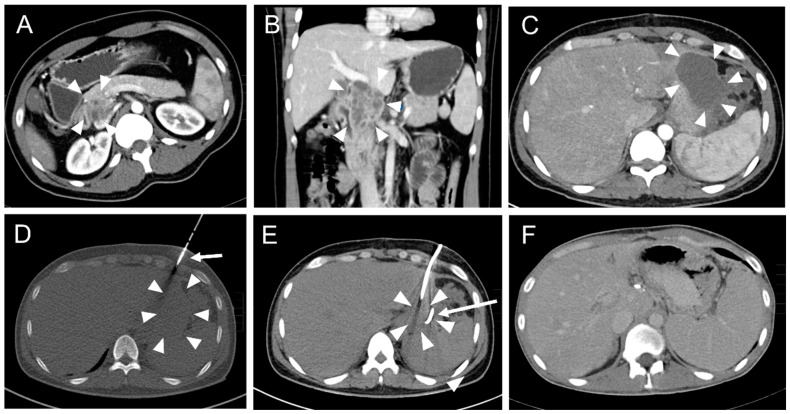
A typical CT fluoroscopy-guided drainage placement procedure after pancreatic surgery. (**A**) A 33-year-old male with a history of abdominal tuberculosis and the formation of an inflammatory pseudotumor in the pancreatic head (arrowheads). (**B**) Coronal reconstruction of the abdominal CT scan illustrates a multicystic mass of the pancreatic head (arrowheads) with close vascular relation to the portal vein and lower vena cava. (**C**) The patient developed subfebrile temperature and left epigastric pain 19 days after pylorus-preserving pancreaticoduodenectomy (PPPD). CT revealed a left paragastric serous fluid collection (arrowheads). (**D**) CT fluoroscopic image (10 mAs tube current). An 8F pigtail drainage was inserted into the fluid collection (arrowheads) with the trocar technique using an anterior intercosto-cartilaginary approach (arrow). (**E**) Post-interventional CT scan. Unenhanced post-drainage (arrow) CT scan showed significantly reduced paragastric fluid collection (arrowheads). Microbiological analysis of the aspirated fluid revealed infection with Serratia marcescens. (**F**) CT follow-up eleven months later showed complete resolution of the fluid collection. Note the increase of spleen size due to portal vein occlusion with beginning cavernous transformation.

Primary placement of the drainage was successful in 165 interventions (Figure 2).

**Figure 3:**
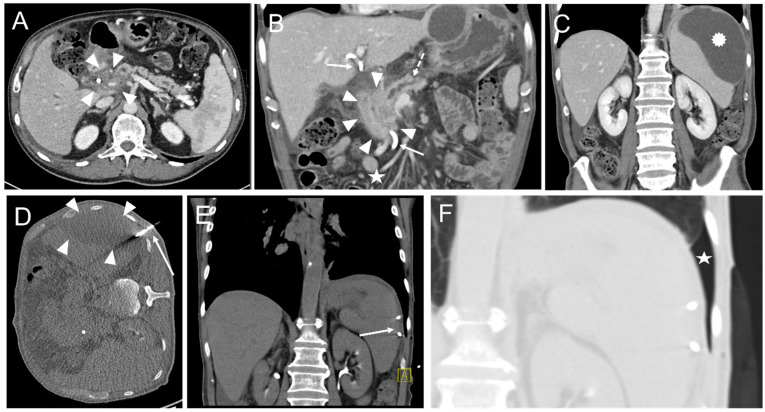
An intervention with a minor complication according to SIR. (**A**) 70-year-old male developing jaundice. CT scan depicts a pancreatic head tumor (arrowheads) with infiltration of the hepatic artery and spread along the portal vein as well as the mesenteric root. (**B**) Infiltration and congestion of the pancreatic duct (dashed arrow), enlarged and roundly configured lymph node metastases (asterisk), and a previously endoscopically inserted drainage (arrows). Arrowheads: pancreatic head tumor. (**C**) An exploratory laparotomy including multiple biopsy samples of the pancreas was conducted. The Patient developed postoperative pancreatitis. Twenty-seven days after surgery a pancreatogenic, perisplenic subcapsular pseudocyst (star) was diagnosed on a CT scan. (**D**) Placement of an 8F drainage (arrow) within the fluid collection (arrowheads) in the right lateral decubitus position, guided by CT fluoroscopy. (**E**) Postinterventional unenhanced control CT scan shows the correct position of the drainage (arrow) and an almost complete disappearance of the fluid collection. (**F**) However, a small self-limiting pneumothorax (star) was observed in the lung window.

**Figure 4:**
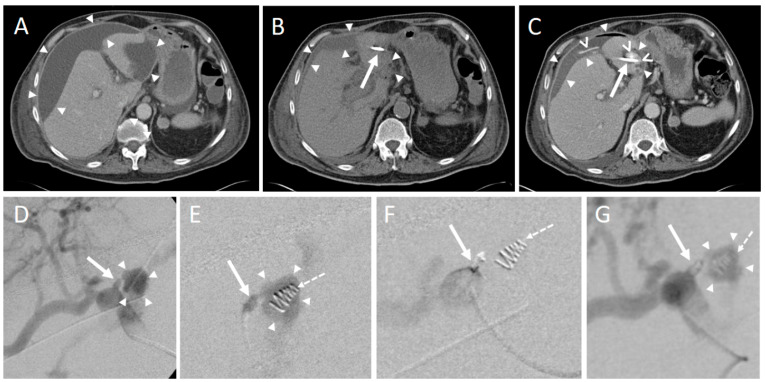
CT-guided drainage placement with a subsequent major complication according to SIR. (**A**) 71-year-old male underwent pancreaticoduodenectomy (PD) due to ductal pancreatic adenocarcinoma. Sixteen days later abdominal revision surgery with hematoma evacuation was performed due to arterial hemorrhage from the hepatic artery and small bowel segment resection due to adhesion ileus. Twenty-seven days after PD a perihepatic subcapsular fluid collection was diagnosed on a CT scan, mainly located at the ventrolateral and medial margins (arrowheads). (**B**) CT fluoroscopy-guided placement of a 10F Flexima^®^ single lumen drainage (arrow; Boston Scientific, Marlborough, MA, USA) within the fluid collection (arrowheads). After placement, a significant reduction of the fluid was observed. (**C**) Contrast-enhanced CT control scan after eleven days revealed an increase of the subcapsular fluid collections mainly next to the right liver lobe (arrowheads). Contrast medium extravasation occurred inferior to the left liver lobe (segment 3) from the left gastric artery in terms of active bleeding from this vessel. Contrast medium extravasation could also be found in the fluid collection (open arrowheads). Arrow: drainage. An angiographic examination (not shown) performed immediately thereafter did not provide the source of the bleeding. (**D**) Repeated angiography after another 6 days showed extravasation of the contrast agent (arrowheads) from the left hepatic artery (arrow). (**E**) Superselective embolization of the bleeding was performed by placement of an 18/4 (Cook Medical, Bloomington, Indiana, USA) Tornado^®^ microcoil in the vessel (arrow) supplying the hemorrhage. However, there was an initial misplacement of the coil (dashed arrow), which came to rest in the adjacent pseudoaneurysm (arrowheads). (**F**) Subsequently, placement of a second 18/4 Tornado^®^ coil was conducted in the vessel neck (arrow; initially misplaced coil: dashed arrow). (**G**) The final angiographic control did not show a remaining contrast agent extravasation. Arrow: Vessel neck with coil; arrowheads: hematoma; dashed arrow: initially misplaced coil. In conjunction with the clinical course, the case was considered to be an arrosion hemorrhage induced by the drainage placement in combination with insufficiency of the biliodigestive anastomosis.

In one intervention, an instant replacement was necessary due to the dislocation of the initial drainage. In one case fluid collection was too small for drainage placement. Complications occurring during or immediately after the intervention were seen in three patients (1.8%). These comprised one major complication (according to SIR criteria) where operative revision was necessary (Table 4, Figure 3 and Figure 4).

Overall, 162 interventions (97.0%) were technically successful.

CRP at baseline (day of the intervention) was (median (25%, 75% quartile)) 17.7 (10.8, 25.2) mg/dL, Leukocytes were 15.8 (12.0; 19.1) × 10^9^/L and Interleukin-6 was 169.0 (98.7, 226.3) pg/dL.

Elevated baseline levels (>0.5 mg/dL) with respect to CRP were observed in 112 interventions (87.5%), regarding Leukocytes (>9.8 × 10^9^/L) in 111 interventions (86.7%) and with regard to Interleukin-6 (>5.9 pg/dL) in 16 interventions (12.5%). In 39 interventions baseline values were not available.

Focussing on the entire intervention DLP (median (25%, 75% quartile)) was 944.5 (730.8, 1177.0) mGy*cm between 2004–2010 and 621.5 (452.8, 878.5) mGy*cm between 2011–2017, corresponding to a significant decrease (*p* < 0.05). Accordingly, all examination parts of the CT intervention differed significantly (*p* < 0.05): Radiation dose in pre-interventional scans was 472 (329, 635) mGy*cm in the years 2004–2010 and 343.0 (215.8, 472.5) mGy*cm in the years 2011–2017. In the years 2004–2010 the DLP of the intra-interventional scan was 116.0 (67.0, 207.0) mGy*cm and 48.5 (26.8, 82.3) mGy*cm in the years 2011–2017. DLP analysis with regard to the post-interventional control scan yielded 316 (255, 389) mGy*cm (years 2004–2010) vs. 245 [169, 331] mGy*cm (years 2011–2017).

**Figure 5:**
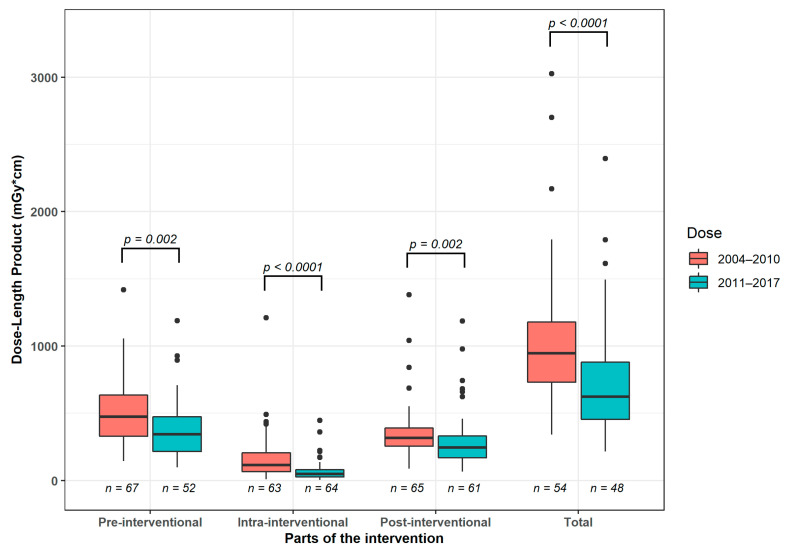
Median radiation dose between 2004–2010 and 2011–2017. Boxplots display elements of the interventional CT scan and the entire procedure.

Comparing both time intervals, CT fluoroscopy was characterized by the most pronounced decrease, corresponding to a reduction of −58.2% of the median value (Figure 5).

### 3.2. Post-Interventional Analysis

We had to exclude four patients from further analysis. Laboratory value data were too sparse or inconsistent for three patients. CT-guided drainage was unsuccessful in one case. Overall, 163 interventions were included in the 60-days post-interventional analysis.

**Figure 6:**
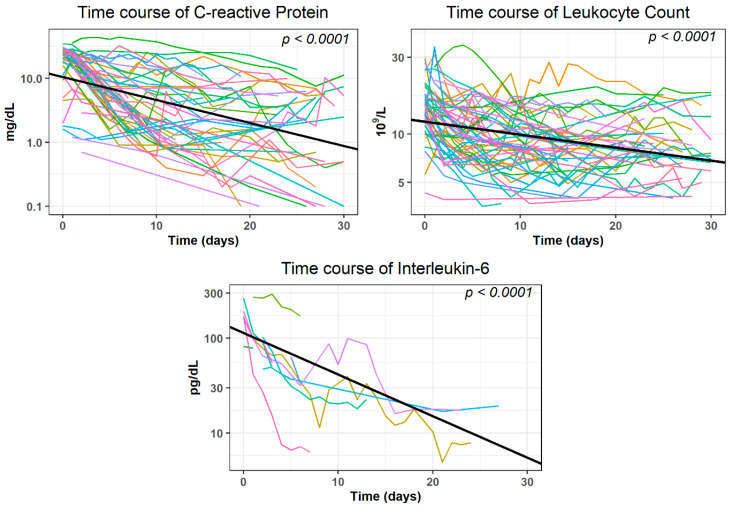
Time course of laboratory parameters within 30 days after the intervention. Subjects had no evidence of additional surgery or complications in their patient records.

A statistically significant (*p* < 0.0001) decrease within 30 days after the intervention was observed in the time course of CRP, Leukocytes, and Interleukin-6 when analyzed with GLMMs in the subgroup of patients where no evidence of further surgical interventions or complications was given (*n* = 120 interventions). The covariate presence of POPF and proof of germs in the drainage fluid were statistically not significant (*p* > 0.220). They were excluded from further GLMM analysis (see Supplementary Table S2 for the results of the final regression models). The decrease of the log-transformed average values was as follows: −0.03593 mg/dL for CRP; −0.00807 × 10^9^/L for Leukocytes and −0.04390 pg/dL for Interleukin-6 (Figure 6).

According to our definition, clinical success (normalization or decrease of 50% of initially elevated inflammatory parameters) was obtained in 98 out of 112 interventions (87.5%) for CRP after (median (25%, 75% quartile)) 6 [4, 8] days, for Leukocytes in 87 out of 111 interventions (78.4%) after 5 [3, 10] days and for Interleukin-6 after 2 [1.3, 3] days in 14 out of 16 cases (87.5%).

Serum amylase levels within 30 days after the intervention only showed a slight increase (log-transformed average value: 0.00171 mg/dL) which was not statistically significant (*p* > 0.05, Supplementary Figure S1).

Table 5 shows the patient success rate among different applied surgical procedures. CRP demonstrated a slightly better response (>82.9%) than leukocytes (>73.7%). The highest rates were obtained in patients with PPPD (88.2% for CRP and 81.8% for Leukocytes, respectively). However, these differences were not statistically significant (*p* > 0.05). A statistical statement about Interleukin-6 or about the distribution of the response rate of these parameters in other resection procedures was not possible due to the small number of cases.

With regard to an unfavorable postinterventional clinical outcome in terms of a required re-operation, surgical revisions had been documented in nine patients (6.8%) due to insufficient fluid collection drainage. This subgroup contained seven patients with PPPD, one with PD, and one with DP. It was not statistically significant (*p* > 0.05). Two of these patients who required re-operation had a POPF and consequently are classified as grade C according to the criteria of Bassi et al. [29].

Microbiological specimens of wound secretions were taken in 149 interventions and were confirmed to be positive in 92 (61.7%) cases. The number of positive results tended (*p* = 0.05) to be lower in interventions with POPF (35 out of 66, 53.0%) than in interventions without POPF (57 out of 83, 68.7%). The most common strains of detected bacteria were Enterococci, diagnosed in 43 patients, and Escherichia asservated in 30 patients. The most frequent pathogenic fungus was Candida, which was the underlying germ in 26 patients. A detailed presentation of the microbiological results is depicted in Supplementary Figure S2.

Comparing infected and non-infected fluid collections, success rates are presented in Table 6. The amount of patients characterized by a significant decrease of laboratory parameter values were higher in the case of an infected fluid collection and highest at 93.3% as far as CRP was concerned.

Given insufficient drainage, three of 57 patients (5.3%) with non-infected fluid-collections had to undergo surgical revision. This corresponds to a lower rate compared to patients with infections, who were subject to a reoperation in 5% (6 out of 92). However, statistical significance (*p* > 0.05) was not reached.

The visual appearance of the drainage fluid was documented in 117 cases (Supplementary Table S3). The fluid collections appeared significantly (*p* < 0.001) more often purulent (54 out of 117 cases, 46.2%). In addition, significantly more germs were positively detected when the drainage fluid was purulent (positive in 40 out of 50 cases (80%) vs. negative in 10 out of 50 cases (20%); *p* < 0.001). However, the biochemically assumed presence of a postoperative pancreatic fistula did not have a significant influence on the appearance of the drainage fluid (*p* < 0.05).

Documented average time to drainage removal was 10.2 ± 8.4 days. Hospitalization after drainage placement was (median (25%, 75% quartile) 19 (11; 32.5) days).

## 4. Discussion

Over the last 30 years, there was an impressive growth of pancreatic resection [30,31,32]. Pancreatic surgery is performed for the treatment of various diseases, mainly malignant tumors and tumor-like lesions. Though, postoperative complication management is demanding. Intraabdominal fluid collections like postoperative pancreatic fistula, abscesses, bile leak, and hemato-seroma following pancreatic resection frequently occur [6,33]. In particular, abscesses and POPF, which itself may be infected, are characterized by significant morbidity and mortality. An early and successful treatment is required [9]. In conjunction with the administration of antibiotics, CT-guided percutaneous drainage has become an increasingly performed alternative for surgery and is the current standard of treatment [8,23,24,26,34,35]. Its main advantages are reduced invasiveness, increased cost efficiency, and the possibility to repeat the intervention within short time intervals if required [16]. In addition, after CT-guided drainage identification of underlying microorganisms is possible, allowing for targeted treatment in case of superinfection.

Our retrospective study comprised a 14 years period in which a large patient cohort was subject to CT-guided percutaneous drainage after pancreatic resection. In our hospital pancreatic surgery was performed in 1824 patients between January 2004 and December 2017. Of these, applying our inclusion criteria yielded 133 patients who received CTD in a total of 167 interventions, an incidence of 7.3%. This agrees with values reported in other studies which range from 4.8% to 12% [23,24]. We analyzed the interventions with regard to the technical and clinical outcomes.

In most patients, one drainage was inserted per intervention. The trocar technique, a single-step procedure, was frequently chosen because it is easier to handle and less time-consuming than the Seldinger technique [36]. A transabdominal access path (74.3%) was preferred compared to a transhepatic (10.2%) or transretroperitoneal access trajectory (15.6%) due to better accessibility of most fluid collections in the supine position. Additionally, it would be uncomfortable for most patients to have the skin exit point of the drain on the back or flank in case a transretroperitoneal access is utilized. Drains of size 8F and 10F were used most frequently, as they can easily be controlled and can also be inserted into smaller collections. Larger drainage diameters imply a greater risk of injury to hollow organs and vessels and are therefore preferably used for suction-irrigation drains such as the van Sonnenberg type.

CT-guided drainage may cause a broad spectrum of complications such as pneumothorax, hemorrhage, sepsis, or death [11,19,20,22]. Complication rates of CTD after pancreatic resection are in a range between 2% and 5% [8,23,25]. The rate of peri-interventional complications in our study was comparatively lower comprising three patients (1.8%) with adverse events, one of them major according to SIR [27]. With regard to the technical success of our study, 97% of interventions were successful. This is in agreement with findings of other authors confirming a high technical success rate in image-guided drainage of abdominal abscesses with a range of 95–100% [13,17,18,23,25].

We defined clinical success as a decrease of elevated inflammation parameters (CRP, leukocyte count, and Interleukin-6) of more than 50% or normalization of these parameters within a 30 day post-interventional interval. Having the above-mentioned definition in mind, clinical success was most frequently noted for the parameters CRP and Interleukin-6 (87.5% of the interventions each), followed by Leukocytes (78.4 %). However, the number of patients for whom Interleukin-6 values could be extracted was comparatively small (*n* = 16). This is probably because its determination is expensive compared to other inflammatory parameters and it is therefore analyzed less frequently.

Clinical success frequently became apparent within the first week (median: after 5 days for Leukocytes, 2 days for Interleukin-6, and 6 days for CRP).

Szgerza et al. [6] found that the three main entities of abdominal fluid collections were unspecified fluid collections, POPFs, and intraabdominal abscesses. Together, they accounted for 45% of surgical complications after pancreatic surgery. We furthermore evaluated the postinterventional serum amylase levels They only showed a non-significant increase. As elevated serum-amylase levels may correlate with the presence of POPF [37,38], this could support the theory of a clinically stable situation under CTD. However, an increase in amylase could also be due to postoperative pancreatitis, for instance. The clinical success rates in our study were independent of the presence of POPF or superinfection of the fluid. This suggests that the curative effect of CTD in purulent abscesses is permanent. In non-purulent fluid collections, on the other hand, the therapeutic effect is the removal of aggressive enzymes that otherwise would digest the anastomoses.

Furthermore, clinical success in our study was reached if a surgical revision related to the intervention could be avoided. The clinical success rate determined by our criteria yielded 93.2% and was in agreement with other authors’ results. Takaki et al. studied 20 patients with percutaneous drainage for POPF and found a success rate of 90%. However, some of the patients in this small collective also underwent other manipulations such as the exchange of occluded catheters. In a large-scale study with 255 patients, Akinci et al. underlined a high efficacy for the percutaneous treatment of intraperitoneal abscesses by image-guided drainage. Primary cure rates, defined as complete healing without the need for repeated drainage, were 68% [39]. However, in contrast to our study, imaging guidance was conducted using fluoroscopy, ultrasound, or CT. A smaller scale study by Asai et al. focusing on an unselected patient cohort comprised 47 patients with 54 drainages in abdominal, retroperitoneal, and pelvic abscesses and attained a clinical success of 94% [11]. A similar clinical success rate of 92% (95 patients with 107 abdominal and pelvic abscesses) was observed by Lagana et al. [18].

As far as our study results are concerned, no significant correlation comparing clinical outcomes and preceding operation techniques were found. This underlines that more extensive surgical techniques did not negatively influence the postoperative course of the patients.

Carrying out a comparison between the time intervals of 2004–2010 and 2011–2017, in our setting a significant (*p* < 0.01) reduction of median DLP could be documented affecting all steps of the whole CT interventions, particularly when CT fluoroscopy was utilized. This may be attributed to different technical developments such as the use of tube current modulation, iterative image reconstruction, and improvement of CT detector and CT fluoroscopy technology [40,41,42]. With the implementation of the 128-slice CT scanners in our department in the second half of the observation period, angular beam detection was used [43]. This allows the X-ray source to be switched off in a specific segment of its rotation. This technique has been shown to be effective in reducing scattered radiation. In addition, the stellar detector was used. Hereby the signal transformation is implemented into the detector itself to reduce electronic noise. With this technique, it is possible to reduce radiation dose by an average of 54% for soft tissue [44].

Another likely aspect is the growing utilization of CT fluoroscopy in general, yielding a training effect for interventional radiologists [45]. Due to the learning curve, a combination of low milliampere of CT fluoroscopy and quick check technique was increasingly used throughout the study period. The latter is the usage of repeated acquisitions of individual CT fluoroscopy images after each change in needle or table position instead of continuous CT fluoroscopy [42]. This helps to reduce the CT acquisition time, directly translating into a reduced patient radiation exposition.

61.7% of the fluid collections tested for germs were positive. This is consistent with the fact that “purulent” was the most common visual impression. In addition, purulent appearing fluids were significantly more likely to be infected. Enterococci, Escherichia coli, Klebsiella pneumonia, Staphylococci, and Streptococci were the most commonly encountered bacteria of wound secretions. Candida was the most prevalent fungal pathogen. The distribution of these pathogens corresponds to the spectrum which is commonly predominant in intraabdominal infections [46,47].

Several limitations of the current study have to be underlined. First, the presented data are retrospective and collected from a single center over a long period of 14 years. Since the beginning of our study period, the therapy and prognosis of pancreatic carcinoma have changed. Adjuvant chemotherapy, for example, was not standard, furthermore, have the chemotherapy-schemes changed over time. However, these and other improvements in therapy are highly relevant considering morbidity and mortality, but not to complication management following operative procedure. Second, there was marked heterogeneity of histology of the underlying tumors as well as the types of pancreatectomy in our patient population. However, this reflects our large university patient spectrum. Third, we based the definition of POPF on the definition of the IPSGF [29] which is based on the amylase value of the fluid collection and must have exceeded the threshold at least once during the 30 days. However, drains are flushed multiple times during the hospital stay. Therefore, the proportion of fistula patients in our study collective may be skewed. Fourth, several patients were retrospectively excluded from the analysis because of missing or incomplete data. All of the aforenamed limitations, especially the retrospective character, can have a relevant influence on the results and lead to bias.

## 5. Conclusions

To conclude, CT fluoroscopy-guided drainage in patients presenting with symptomatic fluid collections after pancreatic resection was highly successful from a technical point of view (97.0%). On the other hand, a fair to good clinical success rate was achieved when evaluating the ratio of patients characterized by a marked decrease of inflammatory blood parameters (CRP: 87.5%, Leukocytes: 78.4%, Interleukin-6: 87.5%) as well as the absence of surgical revisions related to the intervention (93.2%). The complexity of preceding surgical procedures, the presence of a postoperative pancreatic fistula, or a superinfection of the fluid collection did not influence these results. Moreover, the ongoing advancement of CT technology and increased interventional radiologist experience have positively influenced the ongoing reduction of radiation exposure for both patients as well as for interventional radiologists.

In summary, CTD is a safe and successful procedure that can be used to treat symptomatic postoperative intraabdominal fluid collections after pancreatic surgery. Given an elderly and usually multimorbid patient population, this minimally invasive intervention can be particularly advantageous with respect to a temporizing effect avoiding early reoperation.

## Figures and Tables

**Table 1:** Overview of the applied surgery techniques and any additional surgical procedures.

Surgery Technique	Total	Additional Resected Organs or Organ Systems and Performed Surgical Procedures ^2^							
		Hemicolectomy	Splenectomy	Cholecystectomy	Gastrectomy	Adrenalectomy	Hepatectomy	Other Organ Systems	Other Anastomosis
Classic pancreaticoduodenectomy	34 (25.6%) ^1^	3 (2.3 %) ^1^			2 (1.5%) ^1^		1 (0.8 %) ^1^	7 (5.3%) ^1^	2 (1.5%) ^1^
Pylorus-preserving pancreaticoduodenectomy	45 (33.8%) ^1^		1 (0.8%) ^1^				3 (2.3 %) ^1^	6 (4.5%) ^1^	
Distal pancreatectomy	40 (30.1%) ^1^	3 (2.3 %) ^1^	30 (22.6%) ^1^	7 (5.3%) ^1^	3 (2.3%) ^1^	5 (3.8%) ^1^	1 (0.8 %) ^1^	11 (8.3%) ^1^	2 (1.5%) ^1^
Pancreatic segmentectomy/Enucleation/Biopsy sampling	9 (6.8%) ^1^			1 (0.8%) ^1^	1 (0.8%) ^1^			1 (0.8%) ^1^	2 (1.5%) ^1^
Duodenum-preserving pancreatic head resection	5 (3.8%) ^1^			1 (0.8%) ^1^			1 (0.8%) ^1^	1 (0.8%) ^1^	

^1^: Numbers (Percentage), ^2^: if not already included in the original surgical technique. “Other organ systems” comprises locoregional lymphnodes, visceral arteries, and veins as well as various parts of the abdominal viscera and abdominal wall. “Other Anastomosis” includes anastomoses of various intestinal segments.

**Table 2:** Information on the affected anatomical regions, drainages, and techniques in the 167 CTD procedures.

Time from surgery to first intervention (days):	13.5 ± 6.5 (4–30) ^1^
Maximum extension of fluid collection (cm)	8.1 ± 3.0 (3.3–18.0) ^1^
Predominant location of fluid collection	**Count**
subdiaphragmatic	5 (3.0%) ^2^
abdominal wall	4 (2.4%) ^2^
perihepatic	44 (26.3%) ^2^
paragastric	11 (6.6%) ^2^
paracolic	4 (2.4%) ^2^
perisplenic	15 (9.0%) ^2^
peripancreatic	72 (43.1%) ^2^
perirenal	5 (3.0%) ^2^
paraaortal/paracaval	4 (2.4%) ^2^
pelvic	3 (1.8%) ^2^
Drainages per intervention	**Count**
1	127 (76.0%) ^2^
2	39 (23.4%) ^2^
3	1 (0.6%) ^2^
Diameter (French)	**Count**
6	1 (0.7%) ^2^
7.5	3 (2.1%) ^2^
8	66 (46.8%) ^2^
10	53 (37.6%) ^2^
12	14 (9.9%) ^2^
14	3 (2.1%) ^2^
20	1 (0.7%) ^2^
Technique	**Count**
Trocar	151 (90.4%) ^2^
Seldinger	16 (9.6%) ^2^
Access path	**Count**
transabdominal	124 (74.3%) ^2^
transhepatic	17 (10.2%) ^2^
transretroperitoneal	26 (15.6%) ^2^

^1^: Mean value ± standard deviation (range), ^2^: Numbers (Percentage).

**Table 3:** Amount of interventions performed for drainage placement in 133 patients, depending on the surgical technique, presence of POPF, and infection status of the fluid collection in the first intervention.

				Amount of Interventions		
Surgery Technique	Count	Presence of POPF	Positive Proof of Germs in First Intervention	1	2	3
Classic pancreaticoduodenectomy	34	11 (32.4%) ^1^	23 (67.6%) ^1^	25 (73.5%) ^1^	7 (20.6%) ^1^	2 (5.9%) ^1^
Pylorus-preserving pancreaticoduodenectomy	45	18 (40.0 %) ^1^	30 (66.7%) ^1^	38 (84.5%) ^1^	7 (15.5%) ^1^	0 (0.0%) ^1^
Distal pancreatectomy	40	23 (57.5%) ^1^	**14 (35.0%)** ^1^	28 (70.0%) ^1^	10 (25.0%) ^1^	2 (5.0%) ^1^
Pancreatic segmentectomy/Enucleation/Biopsy sampling	9	5 (55.6%) ^1^	5 (55.6%) ^1^	9 (100%) ^1^	0 (0.0%) ^1^	0 (0.0%) ^1^
Duodenum-preserving pancreatic head resection	5	2 (40.0%) ^1^	5 (100.0%) ^1^	3 (60.0%) ^1^	2 (40.0%) ^1^	0 (0.0%) ^1^

^1^: Numbers (Percentage), POPF: postoperative pancreatic fistula, Value in bold indicates significant result in Chi^2^-test.

**Table 4:** Peri-interventional complications according to the Society of Interventional Radiology (SIR).

Type of Complication	Count	Clavien-Dindo Classification
*Minor complication:*		
Small pneumothorax	2 (1.2%) ^1^	I
*Major complication:*		
Hemorrhage	1 (0.6%) ^1^	III a

^1^: Numbers (Percentage).

**Table 5:** The success rate of decreasing laboratory parameters among different applied surgical procedures.

	C-Reactive Protein			Leukocytes			Interleukin-6		
Operation Technique	Elevated (*n*)	Success (*n*, %)	No Success *(n*, %)	Elevated(*n*)	Success (*n*, %)	No Success (*n*, %)	Elevated(*n*)	Success (*n*, %)	No Success (*n*, %)
Classic pancreaticoduodenectomy	31	27 (87.1)	4 (12.9)	31	24 (77.4)	7 (22.6)	7	5 (71.4)	2 (28.6)
Pylorus-preserving pancreaticoduodenectomy	34	30 (88.2)	4 (11.8)	33	27 (81.8)	6 (18.2)	4	4 (100.0)	0 (0.0)
Distal pancreatectomy	35	29 (82.9)	6 (17.1)	38	28 (73.7)	10 (26.3)	4	4 (100.0)	0 (0.0)
Pancreatic segmentectomy/Enucleation/Biopsy sampling	6	6 (100.0)	0 (0.0)	5	4 (80.0)	1 (20.0)	1	1 (100.0)	0 (0.0)
Duodenum-preserving pancreatic head resection	6	6 (100.0)	0 (0.0)	4	4 (100.0)	0 (0.0)	0	0 (0.0)	0 (0.0)
Total	112	98 (87.5)	14 (12.5)	111	87 (78.4)	24 (21.6)	16	14 (87.5)	2 (12.5)

*n*: number; %: percentage.

**Table 6:** Success rate distribution of decreasing laboratory parameters between infected and non-infected fluid collections.

	C-Reactive Protein			Leukocytes			Interleukin-6		
Fluid Collection Infection Status	Elevated (*n*)	Success (*n*, %)	No Success (*n*, %)	Elevated (*n*)	Success (*n*, %)	No Success (*n*, %)	Elevated (*n*)	Success (*n*, %)	No Success (*n*, %)
Infected	60	56 (93.3)	4 (6.7)	59	52 (88.1)	7 (11.9)	8	7 (87.5)	1 (12.5)
Non-infected	39	32 (82.0)	7 (18.0)	38	30 (78.9)	8 (21.1)	4	3 (75.0)	1 (25.0)
Total	99	88 (88.9)	11 (11.1)	97	82 (84.5)	15 (15.5)	12	10 (83.3)	2 (16.7)

*n*: number; %: percentage.

## Data Availability

The data presented in this study are available upon reasonable request from the corresponding author.

## Supplementary Materials

The following supporting information can be downloaded at: https://www.mdpi.com/article/10.3390/diagnostics12092243/s1, Figure S1: Time Course of Serum Amylase. Figure S2: Microbiological results in wound secretions divided into the different strains of bacteria and fungi. Table S1: Indication for pancreatic surgery. Table S2: Parameters of the generalized linear mixed models (GLMM) used in Figure 6. Table S3: Visual appearance of the drainage fluid depending on the infection status and presence of POPF.

## References

[1] Huang J., Lok V., Ngai C.H., Zhang L., Yuan J., Lao X.Q., Ng K., Chong C., Zheng Z.J., Wong M.C. (2021). Worldwide Burden of, Risk Factors for, and Trends in Pancreatic Cancer. Gastroenterology.

[2] Torphy R.J., Fujiwara Y., Schulick R.D. (2020). Pancreatic cancer treatment: Better, but a long way to go. Surg. Today.

[3] Hackert T., Klaiber U., Pausch T., Mihaljevic A.L., Büchler M.W. (2020). Fifty Years of Surgery for Pancreatic Cancer. Pancreas.

[4] Asbun H.J., Moekotte A.L., Vissers F.L., Kunzler F., Cipriani F., Alseidi A., D’Angelica M.I., Balduzzi A., Bassi C., Björnsson B. (2020). The Miami International Evidence-based Guidelines on Minimally Invasive Pancreas Resection. Ann. Surg..

[5] Berry A.J. (2013). Pancreatic surgery: Indications, complications, and implications for nutrition intervention. Nutr. Clin. Pract..

[6] Sierzega M., Kulig P., Kolodziejczyk P., Kulig J. (2013). Natural history of intra-abdominal fluid collections following pancreatic surgery. J. Gastrointest. Surg..

[7] Tjaden C., Hinz U., Hassenpflug M., Fritz F., Fritz S., Grenacher L., Büchler M.W., Hackert T. (2016). Fluid collection after distal pancreatectomy: A frequent finding. HPB.

[8] Cronin C.G., Gervais D.A., Castillo C.F., Mueller P.R., Arellano R.S. (2011). Interventional radiology in the management of abdominal collections after distal pancreatectomy: A retrospective review. AJR Am. J. Roentgenol..

[9] Kawaida H., Kono H., Hosomura N., Amemiya H., Itakura J., Fujii H., Ichikawa D. (2019). Surgical techniques and postoperative management to prevent postoperative pancreatic fistula after pancreatic surgery. World J. Gastroenterol..

[10] Sirinek K.R. (2000). Diagnosis and treatment of intra-abdominal abscesses. Surg. Infect..

[11] Asai N., Ohkuni Y., Yamazaki I., Kaneko N., Aoshima M., Kawamura Y. (2013). Therapeutic impact of CT-guided percutaneous catheter drainage in treatment of deep tissue abscesses. Braz. J. Infect. Dis..

[12] Van Sonnenberg E., Wittich G.R., Goodacre B.W., Casola G., D’Agostino H.B. (2001). Percutaneous abscess drainage: Update. World J. Surg..

[13] Wallace M.J., Chin K.W., Fletcher T.B., Bakal C.W., Cardella J.F., Grassi C.J., Grizzard J.D., Kaye A.D., Kushner D.C., Larson P.A. (2010). Quality improvement guidelines for percutaneous drainage/aspiration of abscess and fluid collections. J. Vasc. Interv. Radiol..

[14] Jiang L., Ning D., Chen X. (2020). Prevention and treatment of pancreatic fistula after pancreatic body and tail resection: Current status and future directions. Front. Med..

[15] Men S., Akhan O., Köroğlu M. (2002). Percutaneous drainage of abdominal abcess. Eur. J. Radiol..

[16] Roberts B.W. (2015). CT-guided Intra-abdominal Abscess Drainage. Radiol. Technol..

[17] Kim Y.J., Han J.K., Lee J.M., Kim S.H., Lee K.H., Park S.H., An S.K., Lee J.Y., Choi B.I. (2006). Percutaneous drainage of postoperative abdominal abscess with limited accessibility: Preexisting surgical drains as alternative access route. Radiology.

[18] Laganà D., Carrafiello G., Mangini M., Ianniello A., Giorgianni A., Nicotera P., Fontana F., Dionigi G., Fugazzola C. (2008). Image-guided percutaneous treatment of abdominal-pelvic abscesses: A 5-year experience. Radiol. Med..

[19] Gee M.S., Kim J.Y., Gervais D.A., Hahn P.F., Mueller P.R. (2010). Management of abdominal and pelvic abscesses that persist despite satisfactory percutaneous drainage catheter placement. AJR Am. J. Roentgenol..

[20] Gervais D.A., Ho C.H., O’Neill M.J., Arellano R.S., Hahn P.F., Mueller P.R. (2004). Recurrent abdominal and pelvic abscesses: Incidence, results of repeated percutaneous drainage, and underlying causes in 956 drainages. AJR Am. J. Roentgenol..

[21] Maher M.M., Gervais D.A., Kalra M.K., Lucey B., Sahani D.V., Arellano R., Hahn P.F., Mueller P.R. (2004). The inaccessible or undrainable abscess: How to drain it. Radiographics.

[22] Nattenmüller J., Filsinger M., Bryant M., Stiller W., Radeleff B., Grenacher L., Kauczor H.U., Hosch W. (2014). Complications in CT-guided procedures: Do we really need postinterventional CT control scans?. Cardiovasc. Interv. Radiol..

[23] Liu T., Sun S., Gao H., Gao Y., Xu Q., Liu X., Miao Y., Wei J. (2020). CT-guided percutaneous catheter drainage of pancreatic postoperative collections. Minim. Invasive Allied Technol..

[24] Sohn T.A., Yeo C.J., Cameron J.L., Geschwind J.F., Mitchell S.E., Venbrux A.C., Lillemoe K.D. (2003). Pancreaticoduodenectomy: Role of interventional radiologists in managing patients and complications. J. Gastrointest. Surg..

[25] Takaki H., Yamakado K., Kuriyama N., Nakatsuka A., Sakuma H., Isaji S. (2017). Percutaneous drainage of pancreatic fistula following pancreatectomy with CT-fluoroscopic guidance. Diagn. Interv. Imaging.

[26] Zink S.I., Soloff E.V., White R.R., Clary B.M., Tyler D.S., Pappas T.N., Paulson E.K. (2009). Pancreaticoduodenectomy: Frequency and outcome of post-operative imaging-guided percutaneous drainage. Abdom. Imaging.

[27] Gupta S., Wallace M.J., Cardella J.F., Kundu S., Miller D.L., Rose S.C. (2010). Quality improvement guidelines for percutaneous needle biopsy. J. Vasc. Interv. Radiol..

[28] Dindo D., Demartines N., Clavien P.A. (2004). Classification of surgical complications: A new proposal with evaluation in a cohort of 6336 patients and results of a survey. Ann. Surg..

[29] Bassi C., Marchegiani G., Dervenis C., Sarr M., Hilal M.A., Adham M., Allen P., Andersson R., Asbun H.J., Besselink M.G. (2017). The 2016 update of the International Study Group (ISGPS) definition and grading of postoperative pancreatic fistula: 11 Years After. Surgery.

[30] Bramhall S.R., Allum W.H., Jones A.G., Allwood A., Cummins C., Neoptolemos J.P. (1995). Treatment and survival in 13,560 patients with pancreatic cancer, and incidence of the disease, in the West Midlands: An epidemiological study. Br. J. Surg..

[31] Michelassi F.A., Erroi F.R., Dawson P.J., Pietrabissa A.N., Noda S.E., Handcock M.A., Block G.E. (1989). Experience with 647 consecutive tumors of the duodenum, ampulla, head of the pancreas, and distal common bile duct. Ann. Surg..

[32] Nakase A., Matsumoto Y., Uchida K., Honjo I. (1977). Surgical treatment of cancer of the pancreas and the periampullary region: Cumulative results in 57 institutions in Japan. Ann. Surg..

[33] Cameron J.L., Riall T.S., Coleman J., Belcher K.A. (2006). One thousand consecutive pancreaticoduodenectomies. Ann. Surg..

[34] Mortele K.J., Girshman J., Szejnfeld D., Ashley S.W., Erturk S.M., Banks P.A., Silverman S.G. (2009). CT-guided percutaneous catheter drainage of acute necrotizing pancreatitis: Clinical experience and observations in patients with sterile and infected necrosis. AJR Am. J. Roentgenol..

[35] Politano A.D., Hranjec T., Rosenberger L.H., Sawyer R.G., Tache Leon C.A. (2011). Differences in morbidity and mortality with percutaneous versus open surgical drainage of postoperative intra-abdominal infections: A review of 686 cases. Am. Surg..

[36] Turan H.G., Özdemir M., Acu R., Küçükay F., Özdemir F.A., Hekimoğlu B., Yıldırım U.M. (2017). Comparison of seldinger and trocar techniques in the percutaneous treatment of hydatid cysts. World J. Radiol..

[37] Furukawa K., Gocho T., Sakamoto T., Tsunematsu M., Haruki K., Horiuchi T., Shirai Y., Yasuda J., Shiozaki H., Onda S. (2021). Intraoperative amylase level of pancreatic juice as a simple predictor of pancreatic fistula after pancreaticoduodenectomy. Pancreatology.

[38] Paik K.Y., Oh J.S., Kim E.K. (2021). Amylase level after pancreaticoduodenectomy in predicting postoperative pancreatic fistula. Asian J. Surg..

[39] Akinci D., Akhan O., Ozmen M.N., Karabulut N., Ozkan O., Cil B.E., Karcaaltıncaba M. (2005). Percutaneous drainage of 300 intraperitoneal abscesses with long-term follow-up. Cardiovasc. Interv. Radiol..

[40] Carlson S.K., Bender C.E., Classic K.L., Zink F.E., Quam J.P., Ward E.M., Oberg A.L. (2001). Benefits and safety of CT fluoroscopy in interventional radiologic procedures. Radiology.

[41] Grosser O.S., Wybranski C., Kupitz D., Powerski M., Mohnike K., Pech M., Amthauer H., Ricke J. (2017). Improvement of image quality and dose management in CT fluoroscopy by iterative 3D image reconstruction. Eur. Radiol..

[42] Paprottka P.M., Helmberger T., Reiser M.F., Trumm C.G. (2013). Computed tomography guidance: Fluoroscopy and more. Radiologe.

[43] Hohl C., Suess C., Wildberger J.E., Honnef D., Das M., Mühlenbruch G., Schaller A., Gunther R.W., Mahnken A.H. (2008). Dose reduction during CT fluoroscopy: Phantom study of angular beam modulation. Radiology.

[44] Christe A., Heverhagen J., Ozdoba C., Weisstanner C., Ulzheimer S., Ebner L. (2013). CT dose and image quality in the last three scanner generations. World J. Radiol..

[45] Rathmann N., Haeusler U., Diezler P., Weiss C., Kostrzewa M., Sadick M., Schoenberg S.O., Diehl S.J. (2015). Evaluation of radiation exposure of medical staff during CT-guided interventions. J. Am. Coll. Radiol..

[46] Brook I. (2008). Microbiology and management of abdominal infections. Dig. Dis. Sci..

[47] Santos S.G., Serufo J.C., Silva R.A., Marra B.A., Reis C.M., Hamdan J.S., Nicoli J.R., Carvalho M.A., Farias L.M. (2003). Microbiologic profile of intra-abdominal infections at Belo Horizonte, Brazil. Am. J. Infect. Control.

