# Supplementary material for: Low-Milliampere CT Fluoroscopy-Guided Percutaneous Drainage Placement after Pancreatic Surgery: Technical and Clinical Outcome in 133 Consecutive Patients during a 14-Year Period
Source: Diagnostics (Basel). 2022 Sep 16;12(9):2243. doi: 10.3390/diagnostics12092243 (PMC9498101; doi:10.3390/diagnostics12092243)
Supplement: Supplementary file 1 [file diagnostics-12-02243-s001.zip › supplementary_tables.pdf]

diagnostics

MDPI

Supplementary Table S1. Indication for pancreatic surgery in 133 patients who underwent CT-fluoroscopy guided percutaneous drainage of abdominal fluid collections after operation.

**Variable**	
Age (years)	64 [55, 72] (19–86) [1]
Sex	
Female	61 (45.9%) [2]
Male	72 (54.1%) [2]
Indications for pancreatic resection	
Tumor lesions—Primary:	95 (71.4%) [2]
Solid exocrine tumors	*49 (36.8%)* [2]
Ductal adenocarcinoma	47 (35.3%) [2]
Acinar cell carcinoma	1 (0.8%) [2]
Solid pseudopapillary neoplasm	1 (0.8%) [2]
Solid non-exocrine tumors	*8 (6.0%)* [2]
Carcinoma of the distal bile duct	6 (4.5%) [2]
Adenoma of the distal bile duct	1 (0.8%) [2]
Squamous cell carcinoma	1 (0.8%) [2]
Solid neuroendocrine tumors (NETs)	*19 (14.3%)* [2]
Cystic lesions	*19 (14.3%)* [2]
Intraductal papillary mucinous neoplasm	11 (8.3%) [2]
Serous cystoadenoma	7 (5.3%) [2]
Cystic ductal adenocarcinoma	1 (0.8%) [2]
Tumor lesions—Secondary:	11 (8.3%) [2]
Renal cell carcinoma	3 (2.3%) [2]
Liposarcoma	3 (2.3%) [2]
Ovarian cancer	2 (1.5%) [2]
Melanoma	1 (0.8%) [2]
Gastrointestinal Stromal Tumor	1 (0.8%) [2]
Endometrial Carcinoma	1 (0.8%) [2]
Tumor-like lesions:	25 (18.8%) [2]
Focal pancreatitis	14 (10.5%) [2]
Pseudocysts	6 (4.5%) [2]
Fatty infiltration	1 (0.8%) [2]
Echinococcosis	1 (0.8%) [2]
Castleman disease	1 (0.8%) [2]
Desmoid	1 (0.8%) [2]
Tuberculosis	1 (0.8%) [2]
Other reasons:	2 (1.5%) [2]
Trauma	1 (0.8%) [2]
Extremely elevated serum tumor markers	1 (0.8%) [2]

[1]: Median [25%, 75% quartile] (range), [2]: Numbers (Percentage)

Supplementary Table S2. Parameters of the generalized linear mixed models (GLMM) used in Figure 6.

	C-reactive Protein			Leukocyte Count			Interleukin-6		
Predictors	***Estimates***	***CI***	***p***	***Estimates***	***CI***	***p***	***Estimates***	***CI***	***p***
(Intercept)	1.02	0.90–1.14	**<0.001**	1.08	1.03–1.12	**<0.001**	2.05	1.83–2.28	**<0.001**
Time (days)	−0.04	−0.04–−0.03	**<0.0001**	−0.01	−0.01–−0.01	**<0.0001**	−0.04	−0.05–−0.04	**<0.0001**
**Random Effects**									
σ^2		0.11			0.01			0.05	
τ_{00}		0.15 Subject ID			0.02 Subject ID			0.13 Subject ID	
ICC		0.57			0.63			0.71	
N		49 Subject ID			55 Subject ID			12 Subject ID	
Observations		417			507			83	
Marginal R^2 / Conditional R^2		0.269 / 0.689			0.117 / 0.672			0.349 / 0.814	

CI: Confidence Interval; R^2: Coefficient of Determination; σ^2: distribution-specific variance; τ00: between-subject-variance; ICC: intraclass correlation coefficient, N: number of subjects.

Supplementary Table S3. Visual appearance of the drainage fluid depending on the infection status and presence of POPF.

Visual appearance [1]		Pancreatic fistula	Proof of germs [2]	
			Positive	*Negative*
Bloody	18 (15.4%)	*Present*	3 (17.6%)	7 (41.2%)
		Not present	3 (17.6%)	4 (23.6%)
Chylus	2 (1.7%)	*Present*	0 (0.0 %)	0 (0.0 %)
		Not present	1 (50.0%)	1 (50.0%)
Purulent	54 (46.2%)	*Present*	11 (22.0 %)	5 (10.0 %)
		Not present	29 (58.0 %)	5 (10.0 %)
Bilious	17 (14.5%)	*Present*	4 (23.5 %)	2 (11.8%)
		Not present	7 (41.2 %)	4 (23.5 %)
Serous	26 (22.2%)	*Present*	5 (20.0 %)	6 (24.0 %)
		Not present	6 (24.0 %)	8 (32.0 %)

[1]: Numbers (Percentage). The group includes all cases where the visual appearance of the drainage fluid was documented (n = 117). Percentages refer to the column values. [2]: Numbers (Percentage). Subgroup in which germ detection and biochemical testing for the presence of a POPF were performed (n = 111). Percentages refer to the values of the corresponding 2 × 2 contingency table. Values in bold indicates significant result in Chi^2-test. POPF: postoperative pancreatic fistula.
