# Supplementary figures and images for: Low-Milliampere CT Fluoroscopy-Guided Percutaneous Drainage Placement after Pancreatic Surgery: Technical and Clinical Outcome in 133 Consecutive Patients during a 14-Year Period
Source: Diagnostics (Basel). 2022 Sep 16;12(9):2243. doi: 10.3390/diagnostics12092243 (PMC9498101; doi:10.3390/diagnostics12092243)

Time course of Serum Amylase

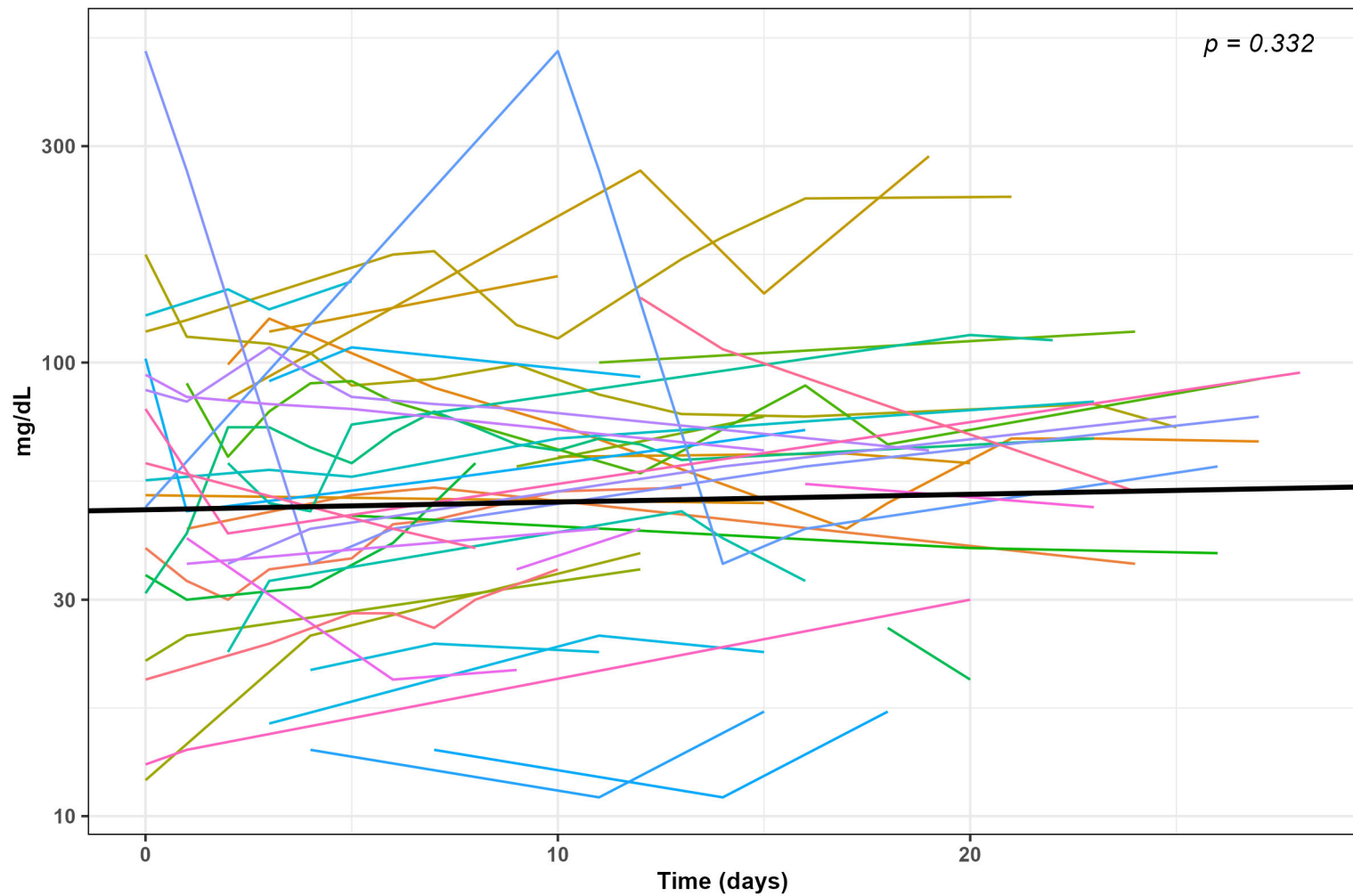

Supplement: Supplementary file 1 [file diagnostics-12-02243-s001.zip › supplementary_figure_S1.pdf]

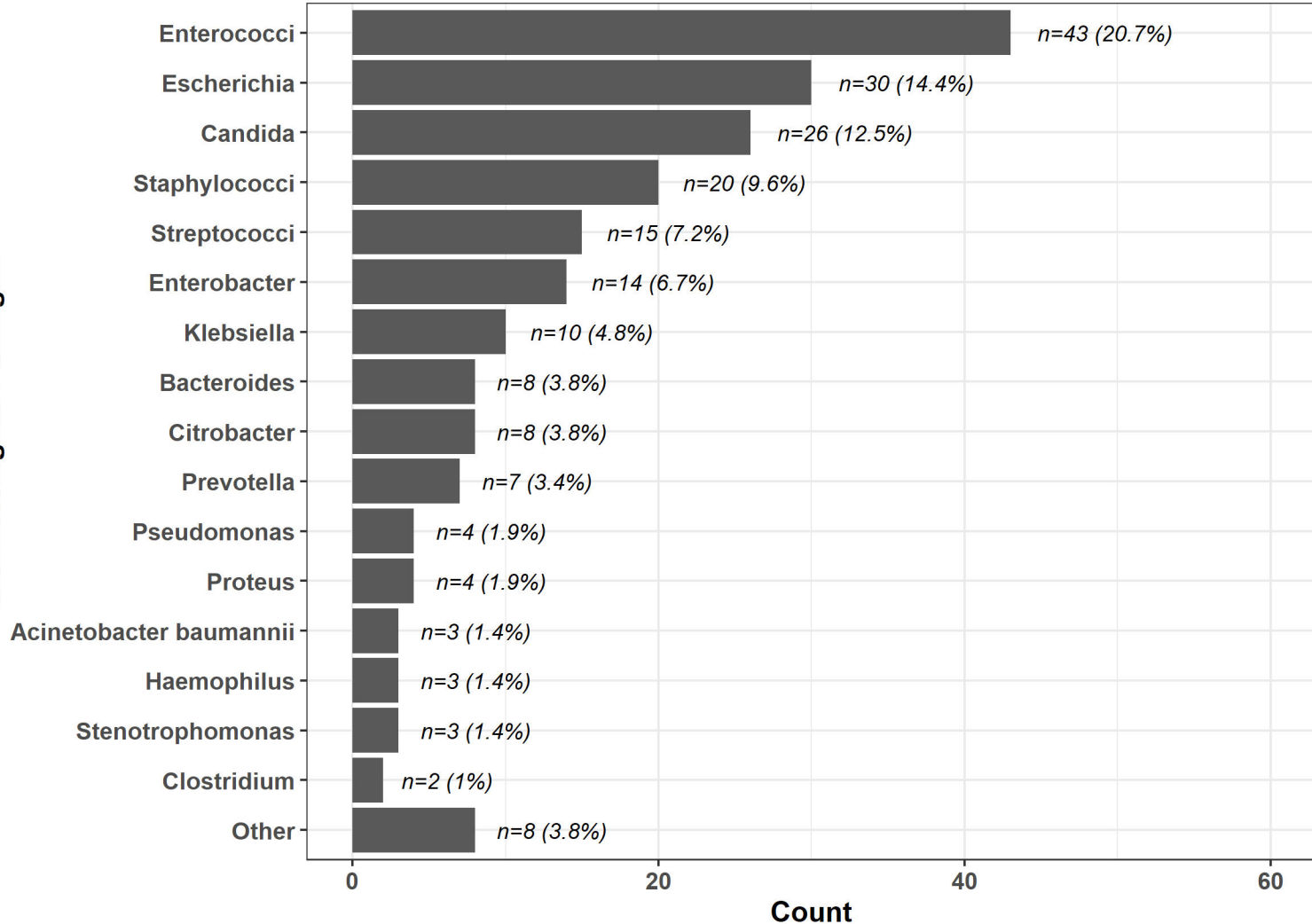

Supplement: Supplementary file 1 [file diagnostics-12-02243-s001.zip › supplementary_figure_S2.pdf]
